# Supplementary material for: The causal role of circulating inflammatory markers in osteoporosis: a bidirectional Mendelian randomized study
Source: Front Immunol. 2024 Jul 18;15:1412298. doi: 10.3389/fimmu.2024.1412298 (PMC11291241; doi:10.3389/fimmu.2024.1412298)
Supplement: Supplementary file 1 [file DataSheet_1.zip › supplementary material/CIM-OSTEOPOROSIS /GCST90274783.csv_leave_one_out.pdf]

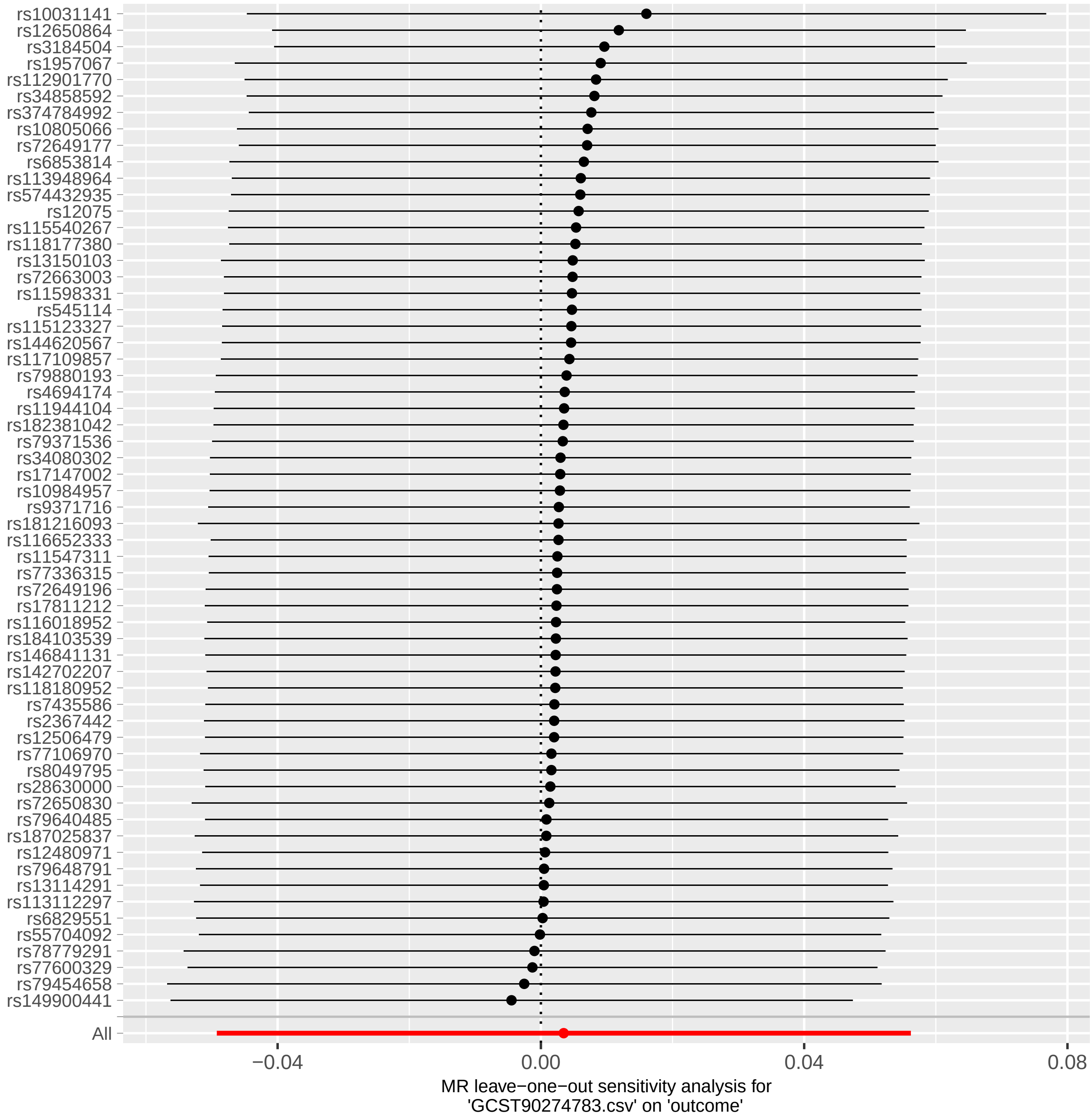
rs10031141
rs12650864
rs3184504
rs1957067
rs112901770
rs34858592
rs374784992
rs10805066
rs72649177
rs6853814
rs113948964
rs574432935
rs12075
rs115540267
rs118177380
rs13150103
rs72663003
rs11598331
rs545114
rs115123327
rs144620567
rs117109857
rs79880193
rs4694174
rs11944104
rs182381042
rs79371536
rs34080302
rs17147002
rs10984957
rs9371716
rs181216093
rs116652333
rs11547311
rs77336315
rs72649196
rs17811212
rs116018952
rs184103539
rs146841131
rs142702207
rs118180952
rs7435586
rs2367442
rs12506479
rs77106970
rs8049795
rs28630000
rs72650830
rs79640485
rs187025837
rs12480971
rs79648791
rs13114291
rs113112297
rs6829551
rs55704092
rs78779291
rs77600329
rs79454658
rs149900441
All
−0.04
0.00
0.04
0.08
MR leave−one−out sensitivity analysis for
'GCST90274783.csv' on 'outcome'
